# Can medical students do anything useful to support the antimicrobial resistance agenda?

**DOI:** 10.1093/jacamr/dlac110

**Published:** 2022-10-14

**Authors:** David Launer

**Affiliations:** University of Oxford Medical School, Medical Sciences Divisional Office, University of Oxford, Level 3, John Radcliffe Hospital, Oxford OX3 9DU, UK

## Abstract

The emergence of antimicrobial resistance (AMR) is a current and future challenge for patients and clinicians, but the role of medical students in combating AMR is not well established. The most important role for students would be supporting the agenda of antimicrobial stewardship (AMS), which is an effective and evidence-based set of actions that mitigate the emergence of resistance. Medical students can be seen as unlikely activists, who have a strong track record of becoming activists for other causes. If they could extend this activist role to AMR/AMS, they could have outsized impact. In order to achieve this, it is imperative that students begin to organize in their own institutions, in collaboration with those working in other disciplines.

## AMR as a challenge for medical students

The increase of antimicrobial resistance (AMR)—against antibacterials in particular—has already caused enormous morbidity and mortality on a global scale. One recent systematic review estimates 1.27 million deaths were directly attributable to resistance in 2019.^1^ This has been termed an ‘overlooked pandemic,’ reflecting how AMR emerges insidiously, often through stubborn infection or prolonged hospital stays, rather than other infectious pandemics, which present more straightforwardly.^2^ AMR is a pandemic that may also be overlooked by medical students, especially as it often receives little attention within the undergraduate curriculum.

There is evidence to show that final-year medical students across the world do not feel equipped to prescribe antimicrobials sustainably, and that their underlying knowledge behind the topic may be lacking.^3^ One recent review of 15 studies of student knowledge, attitudes and perceptions of AMS revealed worrying trends including a widespread belief that antibiotics could be used to treat viral infections such as the common cold.^4^ When asked, the overwhelming majority of students were keen to have more training on antibiotic usage. These findings reflect the views of medical students from diverse settings, including the Global South, but the surveys cited varied widely in their methodology, and investigated questions ranged from local prescribing guidelines to students’ own self-medicating behaviours. To build a more complete view of the current state of medical student ability, it would be useful if a standardized questionnaire could be used that could also allow the value of different educational interventions to be quantified and compared.

Clearly, it is important that these misconceptions are corrected so that students will become prudent antibiotic prescribers when they qualify, and that they are also able to pass on AMR knowledge to future generations of medical students. In the meantime, there may also be roles for students, which are as crucial even before they graduate.

## Possible roles

One possibility is that medical students could contribute to the basic science of drug discovery, which often takes place in institutes that are tightly affiliated to medical schools. This may be a suitable choice for an intercalated bachelor’s or doctoral project, particularly for research-oriented students. These sorts of projects can also be time-consuming and may require spent periods outside of the course. An option that is more feasible for most medical students is the support of AMS. AMS can be seen as an umbrella term for a set of actions that promote using antimicrobials responsibly.^5^ It is already taught to healthcare students and clinicians in the NHS, such as the ‘Start Smart, then Focus’’ approach of prescribing empirically with subsequent review.^6^ It also has an excellent evidence base for reducing the burden of infections from resistant organisms.^7,8^

Students in clinical environments operate at the bottom of a career-long pecking order. They are unpaid, unaffiliated to any particular specialty, and do not have the responsibility (or indeed liability) of their employed colleagues. This may make for advantages as advocates: medical students are able to converse freely with clinicians of all ranks to advocate for stewardship behaviours, in the name of their own education and without seeming to be critical. In particular, being a student is interdisciplinary by nature, so that they may be advocates in many different hospital settings and specialties.

With the magnitude of the AMR threat, it is essential for medical students to further and become stewardship activists. Becoming an activist involves stepping up to form initiatives and lead novel campaigns, rather than working on ones already provided by seniors. It involves breaking the bounds of the curriculum, and possibly going outside the doors of the hospital.

There is a precedent for this kind of work. In the 1970s, the student section of the American Medical Association organized a successful campaign against tobacco smoking on American flights.^9^ The group eventually changed the position of the Association, which itself caused a change in federal law. More recently, Clynch *et al*.^10^ described the creation of a working group at the University of Liverpool aimed at decolonizing the medical curriculum. Similarly, a student at St. George’s Medical School in London wrote a handbook of clinical signs in darker skin, to reflect what was felt to be inadequate teaching at that institution.^11^ It could be argued that AMR shares some of the features that have inspired such student activism; like the healthcare challenges cited, AMR is a problem of injustice. Resistant organisms are most likely to afflict those in low- and middle-income settings, and paradoxically those with least access to antimicrobials.^12^

## Medical students against AMR—starting at home

Why then, are medical students not organizing themselves into an activist movement against AMR? One factor that successful campaigns share is a focus on *local* issues, those which are felt most acutely in one’s own hospital and university. Currently AMR is not conceptualized as being close to home. In a recent systematic review of medical student knowledge, beliefs and attitudes to antibiotic prescribing, there was a disjunction between local and international views across all years of study.^13^ Though the large majority surveyed believed AMR to be a pressing concern, most saw AMR as a global problem, while consistently fewer thought it relevant in their own teaching hospital. Recognizing it at a local level would underline the urgency AMR necessitates without undermining an understanding of AMR as a global socioeconomic crisis.

As with any other campaign, it is vital that medical students organize to act together, and these groups could have myriad roles in the clinical setting and community. This may be in the form of outreach into schools, or more academic functions including lecture series or journal clubs. They would also be a useful base for engaging with educational leaders on AMR teaching and advocating for a greater emphasis on AMS in the curriculum. In all of this, these groups should have a strong online presence as a base for communicating the urgency of AMS. The frontline for any modern activism is social media, and AMR is not an exception.

AMR activism should also be interdisciplinary, and medical students are well-placed to facilitate this. Students would be astute to work with their peers studying the basic or social sciences, including areas like novel antimicrobial development or healthcare policy, both of which have great relevance to the AMR fight. This is true for collaboration with allied healthcare students too, such as pharmacy, nursing and dentistry students who share a frontline role in stewardship. Finally, it may be that a future AMR student movement should try to engage young people regardless of educational area to both educate and hopefully inspire them to work for change themselves.

Forming AMR student groups is a promising but currently unexplored opportunity. Medical students have a real chance to support the AMR agenda by following the example of their peers engaged in other causes like broad ethnic representation in medical curricula. They would be wise to join forces with students in allied healthcare fields and those in other disciplines entirely.
